# Selective block of sensory neuronal T-type/Cav3.2 activity mitigates neuropathic pain behavior in a rat model of osteoarthritis pain

**DOI:** 10.1186/s13075-022-02856-0

**Published:** 2022-07-16

**Authors:** Brandon Itson-Zoske, Seung Min Shin, Hao Xu, Chensheng Qiu, Fan Fan, Quinn H. Hogan, Hongwei Yu

**Affiliations:** 1 Department of Anesthesiology, Medical College of Wisconsin, Milwaukee, WI 53226 USA; 2 Department of Orthopedic Surgery, Affiliated Hospital of Qingdao University, Qingdao, China; 3 Department of Pharmacology and Toxicology, Mississippi University Medical Center, Jackson, MS 39216 USA

**Keywords:** Monosodium iodoacetate, Osteoarthritis, Chronic pain, Dorsal root ganglia, Primary sensory neuron, T-type/Cav3.2 channels, Adeno-associated virus

## Abstract

**Background:**

Peripheral and central nociceptive sensitization is a critical pathogenetic component in osteoarthritis (OA) chronic pain. T-type calcium channel 3.2 (Ca_V_3.2) regulates neuronal excitability and plays important roles in pain processing. We previously identified that enhanced T-type/Ca_V_3.2 activity in the primary sensory neurons (PSNs) of dorsal root ganglia (DRG) is associated with neuropathic pain behavior in a rat model of monosodium iodoacetate (MIA)-induced knee OA. PSN-specific T-type/Ca_V_3.2 may therefore represent an important mediator in OA painful neuropathy. Here, we test the hypothesis that the T-type/Ca_V_3.2 channels in PSNs can be rationally targeted for pain relief in MIA-OA.

**Methods:**

MIA model of knee OA was induced in male and female rats by a single injection of 2 mg MIA into intra-knee articular cavity. Two weeks after induction of knee MIA-OA pain, recombinant adeno-associated viruses (AAV)-encoding potent Ca_V_3.2 inhibitory peptide aptamer 2 (Ca_V_3.2iPA2) that have been characterized in our previous study were delivered into the ipsilateral lumbar 4/5 DRG. Effectiveness of DRG-Ca_V_3.2iPA2 treatment on evoked (mechanical and thermal) and spontaneous (conditioned place preference) pain behavior, as well as weight-bearing asymmetry measured by Incapacitance tester, in the arthritic limbs of MIA rats were evaluated. AAV-mediated transgene expression in DRG was determined by immunohistochemistry.

**Results:**

AAV-mediated expression of Ca_V_3.2iPA2 selective in the DRG-PSNs produced significant and comparable mitigations of evoked and spontaneous pain behavior, as well as normalization of weight-bearing asymmetry in both male and female MIA-OA rats. Analgesia of DRG-AAV-Ca_V_3.2iPA1, another potent Ca_V_3.2 inhibitory peptide, was also observed. Whole-cell current-clamp recordings showed that AAV-mediated Ca_V_3.2iPA2 expression normalized hyperexcitability of the PSNs dissociated from the DRG of MIA animals, suggesting that Ca_V_3.2iPA2 attenuated pain behavior by reversing MIA-induced neuronal hyperexcitability.

**Conclusions:**

Together, our results add therapeutic support that T-type/Ca_V_3.2 in primary sensory pathways contributes to MIA-OA pain pathogenesis and that Ca_V_3.2iPAs are promising analgesic leads that, combined with AAV-targeted delivery in anatomically segmental sensory ganglia, have the potential for further development as a peripheral selective T-type/Ca_V_3.2-targeting strategy in mitigating chronic MIA-OA pain behavior. Validation of the therapeutic potential of this strategy in other OA models may be valuable in future study.

**Supplementary Information:**

The online version contains supplementary material available at 10.1186/s13075-022-02856-0.

## Background

Injection of monosodium iodoacetate (MIA), an inhibitor of glycolysis, into the intra-articular cavity of knee is an established preclinical OA pain model [1]. The pain pathogenesis in MIA-OA has been described as a multifaceted and time-dependent interrelated pathological processes, involving joint damage, structural reorganization of joint afferents, low-grade inflammation, and nerve damage-induced painful neuroplasticity, all contributing to the MIA-OA pain [2].

Ample evidence demonstrates that peripheral and central nociceptive sensitization plays crucial roles in the pathogenesis of OA pain. MIA-OA exhibits PSN and spinal dorsal horn (SDH) neuronal sensitization that resembles neuropathic pain conditions, suggesting mechanistic overlap in these two pathologies [3]. Many studies focus on changes in knee nociceptors and their sensitivity and summate that OA pain is generated and maintained through continuous distinct peripheral nociceptive inputs from the affected joint to their innervating PSNs in DRG [3]. These mechanisms include sensitization of joint afferents by local inflammation and release of inflammatory mediators, bone marrow lesions or micro fractures, subchondral bone remodeling, and increased intra-osseous pressure [4]. However, clinical observation of OA suggest widespread nociceptive sensitization that includes joint afferents and PSNs innervating tissues outside the arthritic joint. For example, most OA patients experience not only pain from the OA joint but also referred pain from the areas remote from the arthritic joint, such that pain severity in OA patients often fails to correlate with the degree of joint pathology [5]. Clinical findings also show that neuropathic pain feature is prevalent in patients with advanced hip and knee OA [6–8], and approximately 20% of patients continue to experience chronic pain after total knee arthroplasty [9]. Recent studies indicate that, in addition to joint damage, DRG-PSN somata morbidity and plasticity contribute to persistent OA neuropathic pain feature [2, 10].

MIA produces peripheral nerve injury responses with PSN somata damage as time progresses [11]. This leads to the dysfunction of peripheral sensory pathways in OA pain beyond exclusive sensitization of joint afferents and associated innervating PSNs [12], and adjacent non-joint innervating PSNs including C-, Aδ-, and Aβ-neurons are also extensively affected, which could induce sensitization of affected joint, dermatomes, and increased nociceptive input to SDH [12, 13]. These comorbidities of joint- and non-joint-innervating PSN sensitization are recognized as an essential pathology for the development and maintenance of OA neuropathic pain-like behavior. Importantly, joint-MIA induced DRG inflammatory and neuronal pathology can maintain pain independent from the damage in the joint in the late pathological stage [14, 15], which may significantly influence the disease course.

Many PSN nociceptive molecules, e.g., voltage-gated ion channels [16, 17], are sensitized in MIA-OA. T-type/Ca_V_3.2 channels (Ca_V_3.2), which are abundantly expressed in PSNs and their axons, determine low-threshold mechanoreceptor function, shape neuronal firing properties, and have been demonstrated in modulating chronic peripheral and central sensitization in various chronic pain conditions [18]. Ca_V_3.2 knockout reduces load-induced OA in mice [19]. Knee MIA induces elevated expression and enhanced function of PSN T-type/Ca_V_3.2 channels [16]. Here, we tested whether reversing enhanced T-type/Ca_V_3.2 channels selectively in DRG-PSNs will mitigate OA pain behavior. We generated AAV-encoding Ca_V_3.2 inhibitory peptide aptamers (Ca_V_3.2iPA2 and 1) that show selective and potent inhibition of Ca_V_3.2 current without affecting ion channel gating properties in our prior studies [20]. Here, we showed that AAV-mediated selective expression of Ca_V_3.2iPAs in the PSNs in vivo produced significant attenuation of evoked and spontaneous pain behavior in both male and female MIA-OA rats. Hyperexcitability of PSNs in MIA rats was normalized after treatment, suggesting that Ca_V_3.2iPA attenuated pain by reversing injury-induced sensory neuronal hypersensitivity.

## Methods

### Animals

Experiments were performed in adult male and female Sprague Dawley rats (5–6 weeks old; 125–150 g body weight) purchased from Charles River Laboratories (Wilmington, MA, USA). Rats were housed in standard 12-h cycle lighting and were allowed ad libitum access to food and water prior to and throughout the experimental protocol. All animal experiments were performed with the approval of the Medical College of Wisconsin Institutional Animal Care and Use Committee in accordance with the National Institutes of Health Guidelines for the Care and Use of Laboratory Animals. All efforts were made to minimize suffering and the numbers of animals used. All survival surgeries were completed in a sterile environment under a surgical microscope in animals anesthetized with isoflurane (2–5%). For tissue harvest euthanasia, animals were deeply anesthetized using isoflurane followed by decapitation with a well-maintained guillotine. The estimated numbers of animals needed were derived from our previous experience with similar experiments [16, 20], and a power analysis was not performed. The numbers of rats used were detailed in the relevant sections or figure legends of the experiments.

### Induction of knee OA

The MIA model of knee OA was induced in isoflurane-anesthetized animals as previously described [21]. After briefly anesthetized with isoflurane (2% *v*/*v*) vaporized in oxygen, rats received a single intra-articular cavity injection of 2 mg MIA (Sigma-Aldrich, St. Louis, MO, USA) in 50-μl sterile 1× phosphate-buffered saline (saline) delivered by a Hamilton syringe (7645-01, Thermo Fisher, Waltham, MA, USA) through the infrapatellar ligament of the right knee, assisted by flexed the knee at a 90° angle. Control rats received intra-articular injection of saline (50 μl).

### Behavior tests

Behavioral tests were conducted between 9:00 AM and 12:00 AM. Experimenters were blinded to the treatment. Animals were habituated in individual test compartments for at least 1 h before each testing [22, 23].

#### Evoked sensory behavior testing

##### Mechanical allodynia (von Frey (vF))

The threshold for withdrawing from normally innocuous mechanical stimulation was assessed by applying the calibrated monofilaments (Patterson Medical, Bolingbrook, IL, USA) to the plantar surface of the hindpaw. Briefly, animals were placed in Plexiglas® boxes on a grid surface to allow filament access to the hindpaw. Beginning with the 2.8-g filament, filaments were applied with just enough force to bend the fiber and held for 1 s. If a response was observed, the next smaller filament was applied, and if no response was observed, the next larger was applied, until a reversal occurred, defined as a withdrawal after a previous lack of withdrawal, or vice versa. Following a reversal event, four more stimulations were performed following the same pattern. The forces of the filaments before and after the reversal, and the four filaments applied following the reversal, were used to calculate the 50% withdrawal threshold [24]. Rats not responding to any filament were assigned a score of 25 g.

##### Mechanical hyperalgesia

Noxious punctate mechanical stimulation (pin test) was performed using the point of a 22-G spinal anesthesia needle that was applied to the center of the hindpaw with enough force to indent the skin but not puncture it. Five applications were separated by at least 10 s, which was repeated after 2 min, for a total of 10 touches. For each application, the evoked behavior was either a very brisk, simple withdrawal with immediate return of the foot to the cage floor, or a sustained elevation with grooming that included licking, chewing, and possibly shaking, which lasted at least 1 s. This latter behavior was referred to as a hyperalgesic response, which is specifically associated with place avoidance [25]. Hyperalgesia was quantified by tabulating hyperalgesia responses as a percentage of total touches.

##### Cooling stimulation (cold)

Acetone was expelled from a syringe attached to PE220 tubing to make a meniscus that was touched to the plantar surface of the hindpaw, such that the drop spread out on the plantar surface of the paw without contact of the tubing to the skin. Each hindpaw was tested 3 times in alternating fashion. Any withdrawal was considered a positive response.

##### Heating plantar test (heat)

Heat threshold of the hindpaw was determined using a Hargreaves device designed for the purpose of identifying thermal sensitivity (Paw Thermal Stimulator System, University Anesthesia Research & Development Group, San Diego, CA, USA). Rats were placed on a temperature-regulated glass platform heated to 30 °C and the hindpaws stimulated with a radiant heat source (50 W halogen bulb) directed through an aperture. The time elapsed from initiation of the stimulus until withdrawal (withdrawal latency) as measured by a series of photocells was measured. Each hind paw was tested 4 times and the withdrawal latency values averaged.

##### Gabapentin injection

Gabapentin (GBP, Sigma-Aldrich) was dissolved in saline immediately before injections and administered intraperitoneally (i.p.) at a volume of 0.5–1.0 ml (final dose of 100 mg/kg body weight). The hindpaw vF and pin tests on the side of ipsilateral to MIA injection were performed at 15-min intervals for 3 h after GBP injection*.*

#### Spontaneous pain behavior testing

##### Conditioned place preference (CPP)

The presence of spontaneous, ongoing aversiveness (i.e., affective dimension of spontaneous pain) was determined by GBP (i.p.)-induced CPP test, as we have previously described [20, 26] with minor modifications. This employs the strategy that the aversive affective dimension of spontaneous pain can be identified by the place conditioning effect of an analgesic administered in a recognizable location. A 3-chamber CPP apparatus was used (Med Associates, St. Albans, VT, USA) in which 2 sliding doors separate the central chamber from the 2 side chambers that have distinct wall stripes and flooring. Animal movement and times spent in each chamber were measured by computer-interfaced infrared photo beams. The CPP procedure consisted of the following phases: (1) On the preconditioning day, rats were allowed to explore both sides of chambers for 15 min, and the time spent on each side was recorded, and the preferred and nonpreferred chambers were identified. The animals showed an extreme predetermined level of preference for one chamber (≥ 70% of total time) at this stage and were excluded for further study. (2) On the conditioning days, place conditioning was conducted using a biased assignment approach to drug pairing, in which saline was paired with the preferred chamber in the morning, and GBP was paired with the non-preferred chamber in the afternoon with a 6-h interval (injections were never paired with the middle grey chamber). Conditioning consisted of intraperitoneal injection and subsequent restriction of the animals within the non-preferred chamber for 45 min. We used a 45-min conditioning time on the basis of tests that GBP (i.p.) maximally reduced mechanical hypersensitivity at 30–60 min after injection. Animals were conditioned for 2 days since 2-day GBP has been reported sufficient to produce CPP in rodent pain model [27–30]. (3) For postconditioning testing, the animals were placed back into the middle grey chamber of the CPP chambers with free access to all chambers for 15 min. The difference score for each animal was calculated, by subtracting the time spent in the saline-paired or GBP-paired chamber before pairing (during preconditioning) from the time spent in each chamber after pairing (postconditioning), and then averaged within each group. Each rat had only a single CPP test 6 weeks after AAV injection. A CPP effect is defined if the animals spend significantly more time in the GBP-paired chamber versus the saline-paired compartment.

##### Weight-bearing asymmetry (Wb)

An Incapacitance tester (Columbus Instruments, Columbus, OH, USA) was used to determine hindpaw weight distribution as previously reported [16]. Rats were placed in an angled Plexiglas chamber so that each hindpaw rested on a separate force plate. The change in hindpaw weight distribution was automatically calculated by the Incapacitance tester (the difference in the amount of weight (g) between the left and right limbs). Essentially, the apparatus calculates an average hindpaw weight distribution over the span of 5 s, and three recordings are taken for each rat. All three recordings are then automatically averaged, and a mean score is displayed. The primary dependent measure was % weight on ipsilateral hindpaw and was determined by the formula: weight bearings (%) = force (g) of right hindpaw/[force (g) of left hindpaw + force (g) of right hindpaw] × 100. A value of less than 50% indicates a reduction in weight borne on the ipsilateral hindlimb.

### AAV constructs

Production, purification, and titration of AAV constructs, including AAV6-GFPCav3.2iPA1 (AAV6-3.2iPA1), AAV6-GFPCav3.2iPA2 (AAV6-3.2iPA2), and AAV6-GFPCav3.2NP (AAV6-NP, inert Cav3.2 peptide from protein N-terminus as control), have been described previously [20]. The titers (GC/ml) of AAV6-3.2iPA1, 2, and AAV6-NP vectors were 2.45 × 10^13^, 3.05 × 10^13^, and 2.26 × 10^13^, respectively. One lot of viral preparation for each vector was used for in vivo experiments.

### Microinjection of AAV vectors into DRG

AAV vector solution was microinjected into the right lumbar (L) 4 and L5 DRG using previously described techniques [22]. Briefly, the surgically exposed intervertebral foramen was slightly enlarged by the removal of laminar bone. Injection was performed through a micropipette that was advanced ~ 100 μm into the ganglion. Rats received L4 and L5 DRG injections of either AAV6-3.2iPA or AAV6-3.2NP (one vector per rat), consisting of 2 μl with adjusted titers containing a total of 2.0 × 10^10^ genome viral particles for each DRG. Injection was performed over a 5-min period using a microprocessor-controlled injector (Nanoliter 2000, World Precision Instruments, Sarasota, FL, USA). Removal of the pipette was delayed for an additional 5 min to minimize the extrusion of the injectate. Following the injection and closure of overlying muscle and skin, the animals were returned to their housing where they remained as the designed experiments required.

### Histology and immunohistochemistry

#### Knee histopathological analysis

The knee joints from the tibia to the distal metatarsal including the tarsal joint were resected and fixed with 10% neutral-buffered formalin for 1 week at room temperature. The fixed specimens were decalcified in Immunocal (Thermo Fisher) for 2 weeks and embedded in paraffin. Sagittal sections of the knee specimens were acquired from the paraffin blocks at 10 μm thickness, deparaffinized, and rehydrated in the order of xylene and series of absolute to 50% alcohol. The rehydrated sections were stained with hematoxylin and eosin (H&E), as described previously [16], for the observation of morphological changes in the articular tissues. Images of knee joint histology were captured using a Keyence BZ-X800 microscope (Keyence Corporation, Itasca, IL, USA).

#### IHC

Our previously described protocol was adopted [20]. In brief, the formalin-fixed, paraffin-embedded (FFPE) tissue sections were deparaffinized, hydrated, and treated by heat-induced antigen epitope retrieval in 10 mM citrate buffer, pH 6.0. Non-specific binding was reduced by incubating the sections for 30 min with a solution of 5% BSA in PBS plus 0.05% Tween20 (PBST). Samples were first immunolabeled with the selected primary antibodies (GFP 1:100, Ca_V_3.2 1:100, CGRP 1:100, and GFAP 1:1000, all described previously) [20] in a humid atmosphere overnight at 4 °C. All antibodies were diluted in PBST, containing 0.05% Triton X-100 and 5% bovine serum albumin (BSA). Normal immunoglobulin G (IgG from the same species as the first antibody) was replaced for the first antibody as the negative controls. The appropriate fluorophore-conjugated (Alexa 488 or Alexa 594, 1:2000) secondary antibodies (Jackson ImmunoResearch, West Grove, PA, USA) were used to reveal immune complexes. Afterward, the sections were rinsed for 10 min in PBST and either processed for a colabeling of primary and secondary antibodies or coverslipped under *Shur/Mount* mounting medium (Thermo Fisher). To avoid false-positive results attributable to cross-binding in double-label combinations, each primary antibody raised in a different species was used for double labeling. To stain the nuclei, 1.0 μg/ml Hoechst33342 (Hoechst, Thermo Fisher) was added to the secondary antibody mixture. The immunostaining was examined, and images were captured using a Nikon TE2000-S fluorescence microscope (El Segundo, CA, USA) with filters suitable for selectively detecting the green, red, and blue fluorescence using a QuantiFire digital camera (Optronics, Ontario, NY, USA). For double labelling colocalization, images from the same specimen but showing different antigen signals were overlaid by digitally merging the captured images.

#### Quantification

Positive antibody immunostaining was defined as the cells having a fluorescence intensity greater than the average background fluorescence plus 2 standard deviations of the cells in a section of negative control (the first antibody omitted) under identical acquisition parameters (*n* = 10 for different markers), identified by Hoechst counterstain at a different wavelength [31]. For quantification of GFP3.2iPA transduction efficiency, every fifth DRG section was selected from the consecutive serial sections (3 to 5 sections for each DRG), and in each selected section, the numbers of GFP labeled cells were counted and transduction efficiency was expressed as the percentage of total neuronal profiles revealed by b3-tubulin (Tubb3) staining [31].

### Whole-cell current-clamp recording of dissociated DRG neurons

1. Dissociated DRG neuronal culture for electrophysiology was performed as described previously [20]. In brief, DRG (L4) from male rats were rapidly harvested from the isoflurane-anesthetized animals and were incubated in 0.01% liberate blendzyme 2 (Roche Diagnostics, Madison, WI) for 30 min, followed by incubation in 0.25% trypsin and 0.125% DNase for 30 min, both dissolved in DMEM/F12 with glutaMAX (Thermo Fisher). After exposure to 0.1% trypsin inhibitor and centrifugation, the pellet was gently triturated in a culture medium containing Neural basal media A (Thermo Fisher) plus 0.5 μm glutamine. Dissociated cells were plated onto 5% laminin-coated glass coverslips (Thermo Fisher), maintained at 37 °C in humidified 95% air and 5% CO_2_, and were studied in 6~8 h after harvest in electrophysiological experiments.
2. Whole-cell current-clamp recording of dissociated DRG neurons was performed as described previously [32], to determine the effects of AAV-mediated Ca_V_3.2iPA2 expression on neuronal excitability. Dissociated small- and medium-sized DRG neurons (< 40 μm in diameter) from saline-injected animals and rats with MIA only, and dissociated DRG neurons with clear GFP expression from MIA rats injected with AAV6-NP or AAV6-3.2iPA2 at 6-week after vector injection were used for recording (*n* = 5 rats per group). For whole-cell current-clamp, patch electrodes had a resistance of 0.7–1.5 MΩ when filled with the pipette solution, which contained the following (in mM): 140 K-gluconate, 5 KCl, 2 MgCl_2_, 0.2 EGTA, 10 HEPES, 4 Mg-ATP, and 0.3 Na^2+^-GTP, 10 Na2-phosphocreatine pH 7.2 with KOH and osmolarity of 296 to 300 mOsm. The extracellular solution contained the following (in mM): 140 NaCl, 4 KCl, 2 CaCl_2_, 2 MgCl_2_, 10 d-glucose, 10 HEPES at pH of 7.4 with NaOH and an osmolarity of 300 mOsm. Whole-cell configuration was obtained in the voltage-clamp mode before proceeding to the current-clamp recording mode. The membrane input resistance was calculated by dividing the end amplitude of steady-state hyperpolarizing voltage deflection by the injected current [16, 33]. Action potentials (APs) were generated by injection of a series of current pulses (100 to 500 pA in steps of 100 pA, 250 ms). The baseline membrane potential had been recorded for 20 ms before the stimulus pulses were injected into the neurons. We defined the resting membrane potential (RMP) as the mean value of the 20 ms pre-stimulus membrane potential in the first trial and the AP rheobase as the minimum depolarizing current required to evoke the first AP. Given the knowledge that nerve injury induces high RMP and low rheobase in DRG neurons [34, 35], the neurons with stable RMP more negative than − 45 mV and overshooting APs (> 80 mV RMP to peak) were used for additional data collection. AP frequency, an indicator of PSN excitability, was determined by quantifying the number of APs elicited in response to depolarizing current injections (250 ms).

### Statistical analyses

All data are presented as mean ± standard deviation of the mean (SEM) and were analyzed with GraphPad PRISM 9 (GraphPad Software, San Diego, CA, USA). The estimated numbers of animals needed for behavior were derived from our previous experience with similar experiments, and the statistical analyses were done afterward without interim data analysis [16]. No data points were excluded. *p* < 0.05 was considered statistically significant. Behavioral changes compared to pre-treatment baseline (BL) and between groups for von Frey, heat, and Wb measurements were generated using repeated measures two-way ANOVA and turkey post hoc for within-group analysis and Bonferroni test for between the groups. Pin and cold test results in discrete numerical data without normal distribution so conservative nonparametric analysis was performed by Friedman’s tests and Dunn post hoc analysis. For comparisons between the groups, the effects of vector injection were characterized by the area under the curve (AUC) analysis. Specifically, the measured behavioral values after the combined injury and vector injection were normalized to the values immediately preceding injury and injection. For the evaluation of vector on established pain (in which vector injection was performed 14 days after nerve injury, BL), the measured values after the vector injection were normalized to the values immediately before the injection (treatment baseline (tBL)) to calculate treatment AUC (tAUC). Calculated tAUCs were compared between vectors by Student’s *t* test for von Frey, heat, and Wb and by Mann–Whitney *U* test pin and cold. The differences in the electrophysiological experiments were compared with one-way ANOVA, two-tailed unpaired *t* test, or Mann–Whitney test, where appropriate.

## Results

MIA-induced knee damages shown in the current study were in accordance with those in our previous report [16]. MIA-treated knees in both male and female animals displayed typical structural joint damage, examined 56 days after MIA knee cavity injection. Knee injected with 2 mg of MIA showed a considerable loss of articular cartilage surrounding the subchondral bone along the joint, combined with reduced chondrocyte numbers, different degrees of bone marrow lesions, and collapse of subchondral bone (Fig. S1). The articular cartilage is incapable of directly generating pain because of both aneural and avascular. Thus, this study did not attempt to correlate MIA-induced cartilage loss, as well as other knee degeneration pathology, to pain behavior, and studies were focused on addressing whether a sensory neuron-selective block of T-type/Cav3.2 activity will migrate neuropathic pain-like behavior.

### Analgesia of established MIA-OA pain by DRG delivery of AAV6-3.2iPA2 in male rats

**Fig. 1:**
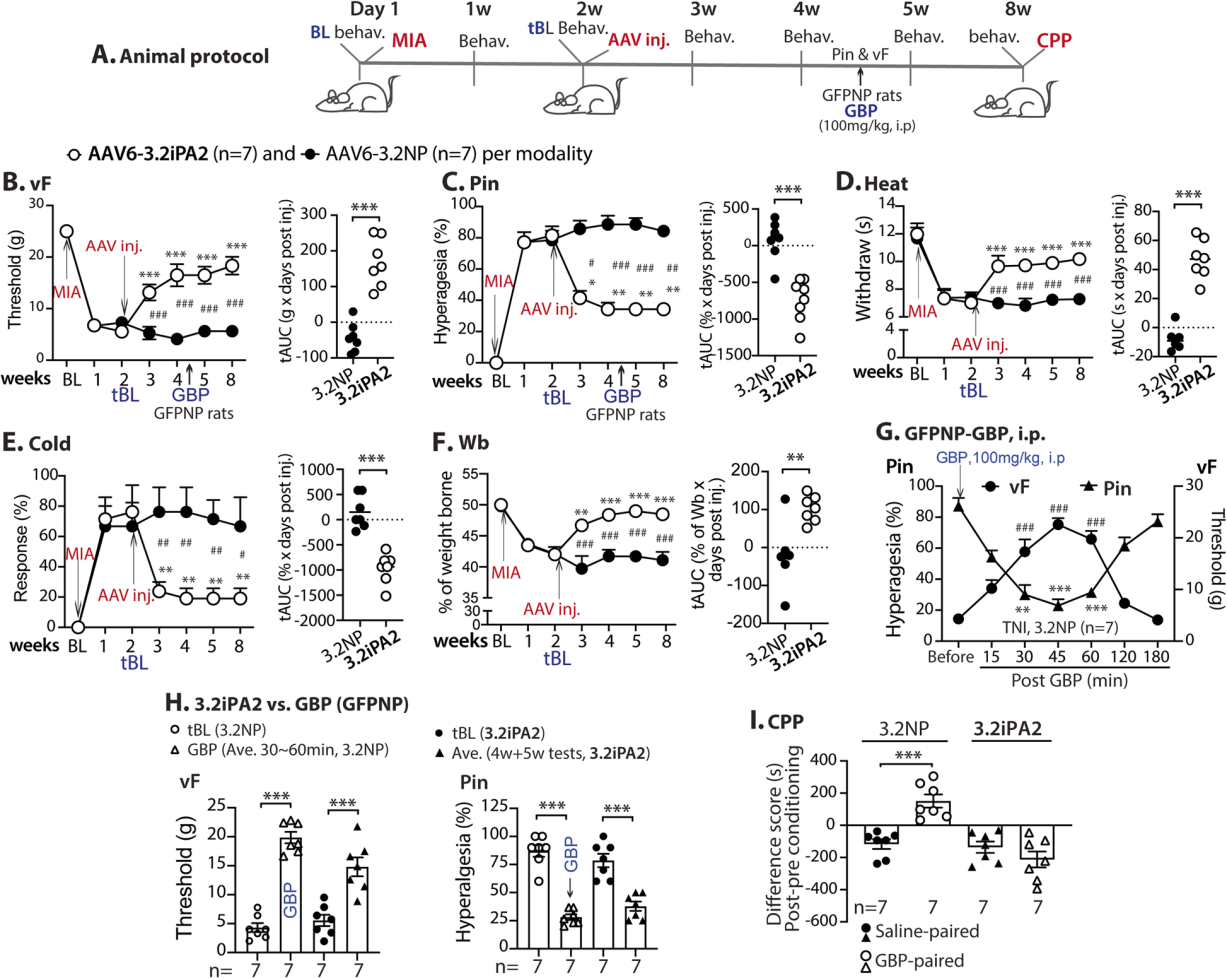
Analgesia of established MIA-OA pain by DRG-AAV6-3.2iPA2 in male rats. Animal protocol is schematically outlined (**A**). The time courses for the group averages of sensitivity to vF, Pin, Heat, Cold, and weight bearing (Wb) before and after DRG injection of either AAV6-3.2iPA2 or AAV6-3.2NP (**B**–**F**); *p < 0.05, **p < 0.01, and ***p < 0.001 for comparisons to tBL within the group and ^#^*p* < 0.05, ^##^*p* < 0.01, and ^###^*p* < 0.001 for comparisons between the groups post-AAV injection. Repeated measures parametric two-way ANOVA for vF, Heat, and Wb followed by Tukey post hoc, and non-parametric Friedman ANOVA for pin and cold tests and Dunn’s post hoc. Right panels of **B**–**F** show tAUCs calculated using the measures 14-day post-MIA and immediate before vector injection as tBL; **p < 0.01 and ***p < 0.001, comparisons of tAUCs between the groups (unpaired, two-tailed Student’s *t* tests for vF, Heat, and Wb and Mann–Whitney *U* tests for pin and cold). **G** The time courses (3 h) of sensitivity to vF and Pin after GBP (100 mg/kg, i.p.) in MIA+AAV-3.2PN rats, performed at the time point as indicated in **A**–**C**; **p < 0.01 and ***p < 0.001 vs. before GBP, repeated measures one-way ANOVA for vF with Tukey post hoc, and ^###^*p* < 0.001 for pin Friedman and Dunn’s post hoc. **H** Comparison of the efficacy between AAV-3.2iPA2 analgesia in MIA and GBP (i.p.) in 3.2NP rats by vF and pin tests, ***p < 0.001, and unpaired two-tailed Student’s *t* test for vF and Mann–Whitney *U* test for Pin. **I** Results of the CPP difference scores (seconds) of saline-paired chamber and the GBP-paired chamber between AAV-3.2iPA2 and AAV-3.2NP, ***p < 0.001 (unpaired, two-tailed Student’s *t* test)

Ca_V_3.2iPA2 (3.2iPA2) and Ca_V_3.2iPA1 (3.2iPA1) are potent Ca_V_3.2 inhibitory peptides derived from intrinsically disordered domains of Cav3.2 protein [20]. Our prior data showed that targeted DRG-PSN expression of AAV6-3.2iPA2 (and 3.2iPA1) was effective in the relief of pain behavior in a rat model of tibial nerve injury (TNI) neuropathic pain without affecting normal pain thresholds in naïve animals [20]. To broaden the testing of analgesic effects across different pain models, we set up experiments to address whether DRG delivery of AAV6-3.2iPAs is also effective in mitigating MIA-OA pain behavior. Specifically, the experiments were first aimed at testing whether AAV-mediated 3.2iPA2 expression selectively in the DRG-PSNs was effective in reversing the neuropathic pain-like behaviors, including evoked and spontaneous or ongoing pain, as well as weight-bearing asymmetry, following MIA knee injection. In the experimental design (Fig. 1A), the sensitivity to mechanical and thermal hindpaw cutaneous stimulation, as well as asymmetric hindlimb weight bearings on the injured limb loading, were assessed at baseline (before MIA) and weekly after MIA knee injection for 2 weeks before DRG delivery of AAV. After behavior tests at the 14th day following MIA, rats were randomized to receive intraganglionic injection of either AAV6-3.2iPA2 (treatment) or AAV6-3.2NP (control) into the ipsilateral L4/L5 DRG, since these DRG innervate knee and sciatic nerves, and show evidence of injury and increased T-type/Cav3.2 activity in knee MA-OA animals [16, 36]. Thereafter, sensory behavior evaluation was performed on a weekly basis for an additional 6 weeks. Single-dose GBP (100mg/kg, i.p.) was administrated to the rats between the 4th and 5th weeks after AAV6-3.2NP vector (control) injection as a positive control to compare antinociceptive effectiveness between the AAV6-3.2iPA2 treatment and GBP. The GBP-CPP test was performed in both AAV6-3.2iPA2 and AAV6-3.2NP groups after the observation course to evaluate spontaneous affective pain between the treatment and the control groups. The sensory behavior testing before vector injection at the 14th day after MIA knee injection was used to evaluate pain behavior development induced by MIA knee injection and also as a treatment baseline (tBL) to compare the efficacy post vector injection. After which, the tissues were harvested for IHC characterization of transgene and target gene expression and for current-clamp recordings of PSN excitability.

The results showed that all rats received 2 mg MIA knee injection established significant ipsilateral pain behavior 2 weeks after MIA, tested on the plantar surface of the arthritic hindpaw, which included reduced threshold for withdrawals from mild mechanical stimuli (vF testing), more frequent hyperalgesic-type responses after noxious mechanical stimulation (pin testing), more frequent withdrawals to heat and cold stimuli (acetone stimulation), and increased arthritic hindlimb weight bearing asymmetry throughout the course of the study. These behaviors persisted in arthritic hind limbs after injection of the control AAV6-NP during the 6 weeks post-injection observation course. In contrast, MIA rats injected with AAV6-3.2iPA2 showed progressive and persistent reversal of these behavioral changes (Fig. 1B–F). Interestingly, MIA-induced changes in weight distribution in the arthritic hindpaw paralleled changes in hindpaw mechanical and thermal sensitivity, suggesting neuropathic components responsible for weight bearing asymmetry after 2 weeks of inflammation stage. GBP (i.p.) applied between the 4th and 5th weeks in MIA+AAV6-3.2NP rats substantially reversed mechanical hypersensitivity at 30 to 60 min after GBP, but animals were fully mechanically hypersensitive again 3 h after injection (Fig. 1G). The average (4w+5w) allodynia (vF) and hyperalgesia (Pin) in MIA+AAV-3.2iPA2 group (~ 60% reversal of tBL) were comparable to the averaged (30 + 45min) values post-intraperitoneal administration of GBP in AAV6-GFPNP group (Fig. 1H). Spontaneous pain is a feature of knee MIA-OA pain, and CPP is the method of testing that has been shown to indicate the presence of spontaneous pain [37]. Using a biased CPP paradigm [38], the effect of AAV-3.2iPA2 treatment on spontaneous pain was evaluated. None of the animals in both groups was excluded from the study because of their extreme baseline preference/avoidance for a chamber [38]. A significant CPP effect was observed in the MIA rats injected with AAV6-3.2NP while there was no significant difference in the times spent in the initially nonpreferred chamber during baseline vs. test period in AAV-3.2iPA2-treated MIA animals (Fig. 1I). This indicates that MIA rats treated with AAV-3.2iPA2 relieved ongoing or spontaneous pain amenable to GBP treatment.

### AAV-mediated Ca_V_3.2iPA2 expression in vivo

**Fig. 2:**
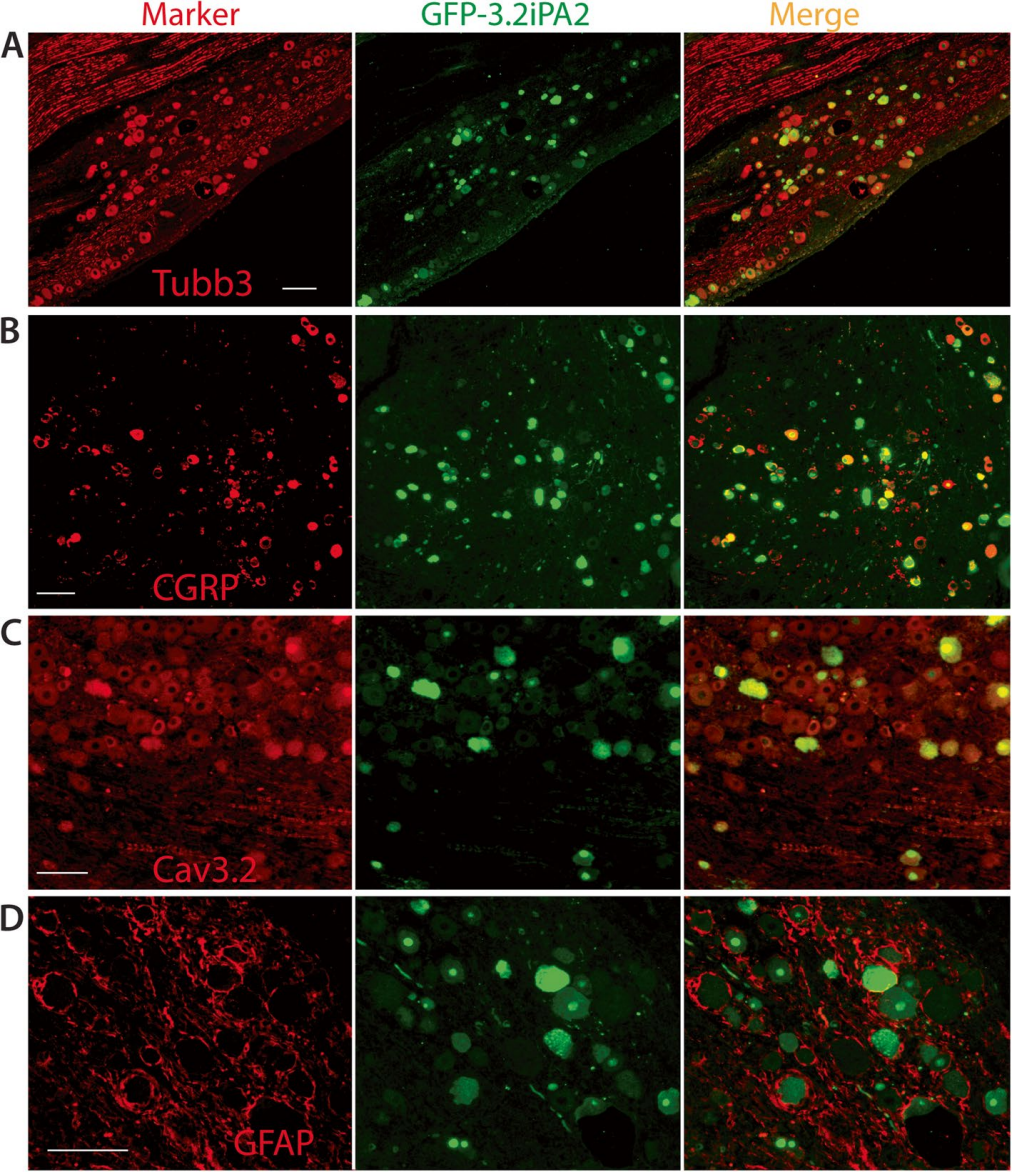
IHC characterization of 3.2iPA2 expression in vivo (male). Representative IHC montage images show GFP-3.2iPA2 expression (green), colabeled with Tubb3 (**A**, red), CGRP (**B**, red), Ca_V_3.2 (**C**, red), and GFAP (**D**, red). GFP-3.2iPA2 signal (green) is not detected in GFAP-positive glial cells (**D**, red). Scale bar, 100 μm for all images

The in vivo transduction rate for AAV6-3.2iPA2 6 weeks after vector injection was determined by IHC. The 3.2iPA2-positive neurons (GFP) comprised 41 ± 10% (824 out of 1986 total neuronal profiles), positive for pan-neuronal marker β3-tubulin, *n* = 3 DRG, five sections per DRG, which were selected as every fifth section from the consecutive serial sections. Transduced DRG neurons included the full-size range of the PSNs that also expressed Ca_V_3.2 (Fig. 2A–C), and GFP3.2iPA2 signals were not detected in the GFAP-positive DRG perineuronal glia cells (Fig. 2D).

### Analgesia of DRG-AAV6-3.2iPA2 treatment in female MIA-OA rats

**Fig. 3:**
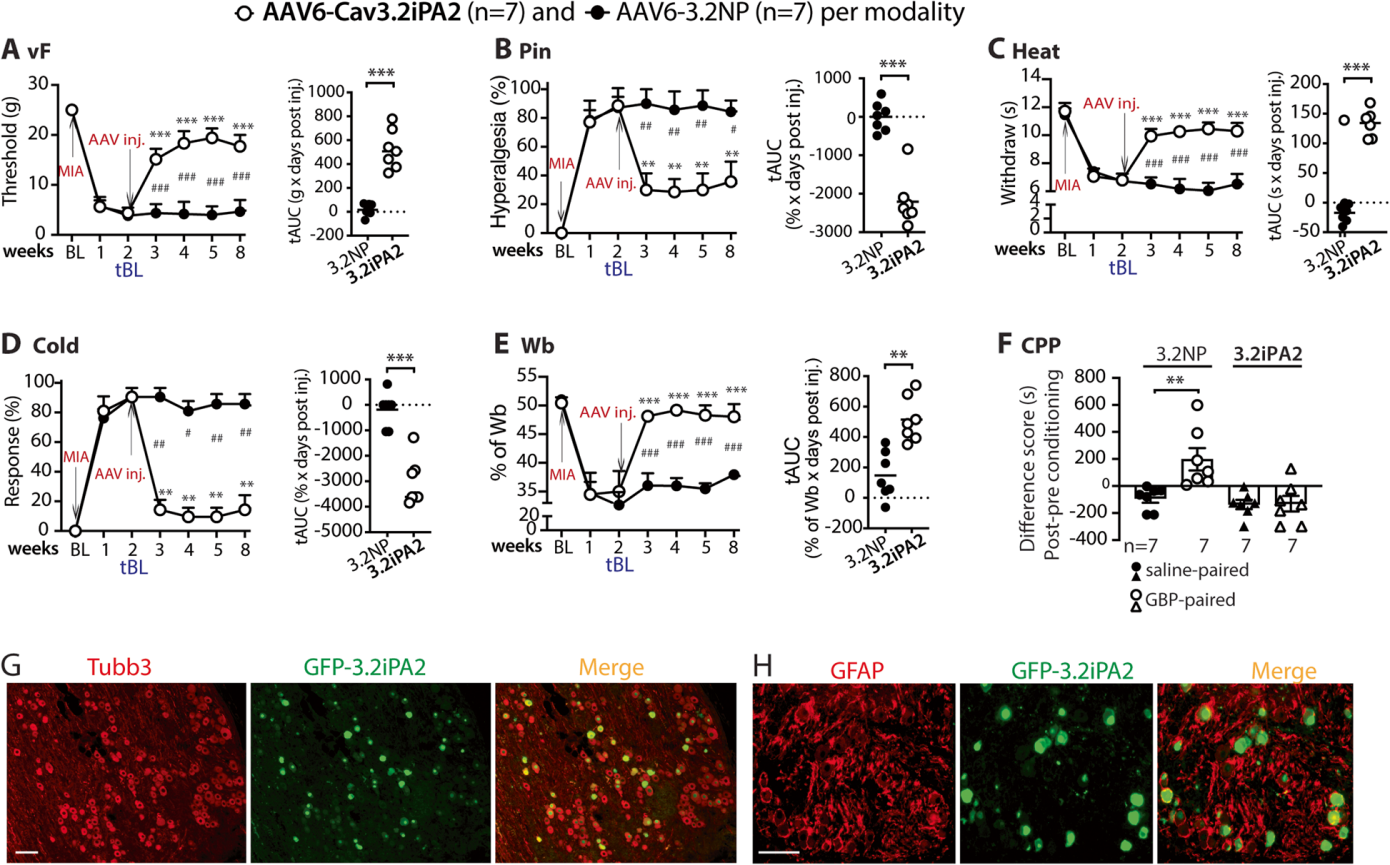
Analgesia of DRG-AAV6-3.2iPA2 injection in female MIA rats. Analogous figures to Fig. 1 show significant analgesia after DRG delivery of AAV6-3.2iPA2 to the established MIA pain behavior in female rats. **p < 0.0.1 and ***p < 0.001 for comparisons to the treatment baseline (tBL) within the group and ^#^*p* < 0.05, ^##^*p* < 0.01, and ^###^*p* < 0.001 for comparisons between the groups post-AAV injection (**A**–**E**). Repeated measures parametric two-way ANOVA for vF, Heat, and Wb followed by Tukey post hoc, and non-parametric Friedman ANOVA for pin and cold tests and Dunn’s post hoc. Right panels of **A**–**E** show tAUCs calculated using the measures 14-day post MIA and before vector injection as tBL; **p < 0.0.1 and ***p < 0.001, comparisons of tAUCs between the groups (unpaired, two-tailed Student’s *t* tests for vF, Heat, and Wb, and Mann–Whitney *U* tests for pin and cold). CPP difference scores (s) of saline-paired chamber and the GBP-paired chamber between AAV-3.2iPA2 and AAV-3.2NP, **p < 0.01 (unpaired, two-tailed Student’s *t* test) (**F**). Representative IHC montage images of GFP3.2iPA2 with Tubb3 show neuronal expression profile after AAV-3.2iPA2 injection (**G**). GFP-3.2iPA2 signal (green) is not detected in GFAP-positive glial cells (**H**, red). Scale bar, 100 μm for all images

Sex differences exist in experimental and clinical pain and in response to pain interventions [39]. We therefore next tested whether DRG-AAV6-3.2iPA2 treatment is also effective in attenuating pain behavior and weight-bearing asymmetry induced by MIA knee injection in female animals, using the same animal protocol as that in male rats (Fig. 1A). Specifically, vector injection was performed 2 weeks after right knee MIA, both evoked mechanical and thermal nociception, arthritic limb weight-bearing asymmetry, and GBP-CPP were evaluated. The same batch preparation of AAV6-3.2iPA2 tested in the male rats was used. The results (Fig. 3A–E) showed that the female MIA-OA rats displayed comparable phenotypic development of hypersensitivity after induction of MIA-OA to male rats and that both evoked mechanical/thermal hypersensitivity, arthritic limb weight-bearing asymmetry, and GBP-CPP responses were substantially normalized after AAV6-3.2iPA2 treatment, showing analgesic effects comparable to those in male animals. IHC on the DRG sections from the female MIA rats 6 weeks after intraganglionic injection of AAV6-3.2iPA2 also revealed a comparable GFP-3.2iPA2 expressive profile to the male rats (Fig. 3F, not quantified). Thus, although male and female groups were not directly compared, a sexual dimorphism was not apparent for either pain behavior phenotypes after MIA or for the response to DRG-3.2iPA2 treatment in this study.

Together, these findings suggest that AAV6-mediated, DRG-targeted Cav3.2iPA2 treatment has analgesic efficacy in normalizing the established peripheral hypersensitivity displaying as evoked and spontaneous ongoing pain in a rat model of MIA-induced OA-pain, effective in both males and females. Additionally, the arthritic limb weight-bearing asymmetry was also significantly normalized. A sexual dimorphism seemed not apparent for both pain behavior phenotypes after MIA knee injection and in responsivity to DRG-3.2iPA2 treatment. Notably, sustained analgesic effectiveness was observed for several weeks during the testing course of this study. Sustained analgesic effectiveness will require evaluation in future studies.

### Reversal of MIA-induced PSN hyperexcitability of MIA-OA pain by AAV6-Ca_V_3.2iPA2 treatment (male rats)

**Fig. 4:**
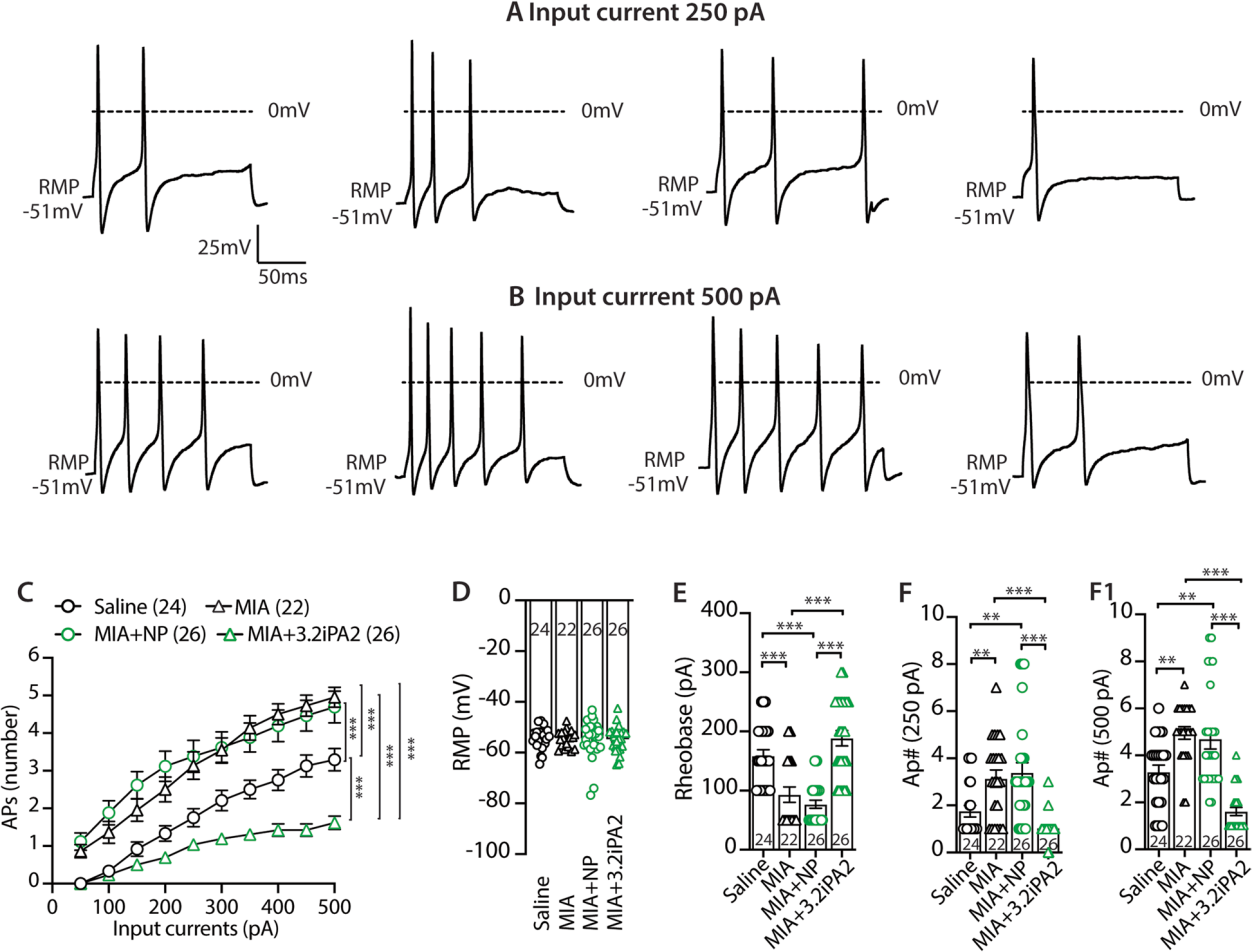
Current-clamp analysis of AAV6-3.2iPA2 transduction on PSN excitability (male). Representative AP traces elicited by 250 ms depolarizing current of 200 pA (**A**) and 500 pA (**B**) (same cells) from rest membrane potential (RMP) were recorded on PSNs dissociated from the rats of saline, MIA, and GFP-expressing neurons in MIA rats treated with AAV6-3.2NP or AAV6-3.2iPA2, as indicated. Comparison of responses (number of APs evoked by a 250-ms stimulus) for the PSNs in different groups across a range of step current injections from 100 to 500 pA (**C**); ***p < 0.001, two-way ANOVA of main effects of the groups with Bonferroni post hoc. Scatter plots with bars show analyses of the RMP (**D**), rheobases (**E**), and AP numbers evoked by input current at 250 pA (**F**) and 500 pA (**F1**) from RMP, respectively. The number in each group is the number of analyzed neurons per group. ** and *** denote *p* < 0.01 and < 0.001, respectively. One-way ANOVA and Turkey post hoc

Ca_V_3.2 channels contribute to nociception by driving burst firing of action potentials (AP) in PSNs, implying that activation of Ca_V_3.2 channels could underlie OA pain-related increase in DRG-PSN hypersensitivity. Our previous study showed that Ca_V_3.2iPA attenuation of neuropathic pain in a TNI model is associated with reversal of injury-induced neuronal hypersensitivity [20]. Therefore, we next test whether analgesia of DRG-3.2iPA treatment in MIA-OA pain is associated with suppression of PSN hyperexcitability. Although MIA results in DRG with co-mingled injured and uninjured axons, nerve injury can induce an increase of voltage-gated ion channel activity in both injured neurons and adjacent intact neurons, leading to similar electrophysiological changes and increased discharge frequency in damaged and neighboring intact DRG neurons [40]. We therefore recorded from randomly chosen small- and medium-sized neurons in the cultures from dissociated L4 DRG. Sensory neurons (small/medium, < 40 μm in diameter) [41] dissociated from DRG of saline-injected rats and MIA rats without treatment, GFP-expressing neurons injected with either AAV6-GFPNP or AAV6-3.2iPA2, were used for recording. Transduced neurons were identified by GFP fluorescence, and excitability was evaluated by measuring the rheobase and the repetitive firing during 250-ms current injection steps. The effect of AAV6-3.2iPA2 expression on the DRG-PSN repetitive firing properties was assessed by applying a series of 250-ms current injections to the DRG dissociated neurons. Although RMP in the neurons from MIA did not differ from saline animals as reported previously [42], the frequency of APs evoked by progressively greater depolarizations in the recorded neurons from MIA rats was significantly increased, compared to saline controls, as previously reported [43]. The increased PSN excitability in MIA rats was normalized in the transduced neurons after AAV6-3.2iPA2 treatment, whereas AAV6-NP-transduced neurons had no effect (Fig. 4). Together, these findings indicate that the reversal of MIA-induced sensory neuronal hyperexcitability [16] by Cav3.2iPA2 may contribute to its attenuation of neuropathic pain behaviors.

### Analgesia of DRG AAV6-3.2iPA1 treatment in MIA-OA rats in male rats

Analogous to the analgesic effects of AAV-3.2iPA2, DRG injection of AAV-3.2iPA1 resulted in a comparable effectiveness of pain relief against both evoked mechanical and thermal hypersensitivity, spontaneous pain behavior, and asymmetric hindlimb weight bearings (Suppl. Fig. 2). A similar in vivo transduction profile to AAV6-3.2iPA1 in male rats was observed for intraganglionic injection of AAV6-3.2iPA1 (not quantified). We did not test the effects of 3.2iPA1 on MIA-induced PSN hyperexcitability. However, reversal of MIA-induced PSN hyperexcitability by AAV6-3.2iPA1 treatment is expected, as we have shown in our previous report that 3.2iPA1 suppresses PSN hyperexcitability following TNI neuropathic pain [20].

## Discussion

Our data affirm that AAV-Ca_V_3.2iPA mediated PSN-selective block of T-type/Ca_V_3.2 activity significantly mitigates evoked hindpaw hypersensitivity in knee MIA-OA rats. Moreover, the PSN-targeted block of T-type/Ca_V_3.2 channels normalizes the arthritic limb weight-bearing asymmetry and the aversiveness of ongoing pain (i.e., affective dimension of spontaneous pain). These data suggest that PSN injury in somata is a significant driver for MIA-OA pain. Dysfunction of T-type/Ca_V_3.2 seems to be a pathological requisite that contributes to the development and maintenance of neuropathic pain-like behavior in MIA-OA, and block of the firing of action potentials through inhibition of T-type/Ca_V_3.2 in PSNs may be an effective means of mediating analgesia in MIA-OA. This is consistent to a previous report showing that DRG stimulation, which suppresses sensory neuronal excitability [44], alleviates pain behaviors in rat MIA-OA models [45]. Additionally, Ca_V_3.2iPA1/2 inhibit both Ca_V_3.2 and Ca_V_3.1 currents (but not Cav3.3 current), demonstrated in our prior study [20]. Thus, the multipronged feature of Cav3.2iPA1/2 with combined Ca_V_3.2 and Ca_V_3.1 inhibition restricted to PSNs may provide an additional analgesic advantage because both are known nociceptive hubs [18, 46].

In OA, the early stage of pain generation of neuropathic components is conceivably initiated at the injury side due to sensitized PSNs innervating joint, by which the specialized sensory afferents detect joint damage-derived chemical, mechanical, or thermal algogenic stimuli. This rationalizes the local intervention of sensitized sensory afferents, i.e., pain arising from the site of injury [47, 48]. However, recent advances have highlighted that pain-related sensory innervation in OA pain progressively induces morbidity in the PSN somata in addition to arthritis knee pathology [11]. DRG (and spinal cord) changes have been recently recognized to be associated with pain chronicity in OA, especially at the advanced stage of disease [49]. More recent literature has documented profound molecular and cellular changes in the DRG of various OA models, including changes in neuronal excitability [50], intense proliferation and activation of microglia that mediate neuroinflammation, and neuronal damage [15, 51–53], transcriptomic upregulation of the genes for neuroimmune interactions [54], sensitized PSN nociceptive voltage-gated ion channels [16, 55] and mechanosensitive ion channels [42], and upregulated chemokine and cytokine production [56], all contributing interrelatedly in driving continuous neural sensitization after cessation of joint inflammation.

Several mechanisms may determine that DRG-PSN morbidity in OA may not be limited exclusively to knee-innervating PSNs. Primarily, although PSN somata in DRG are anatomically isolated from each other and are not synaptically interconnected, most DRG-PSNs are transiently depolarized when axons of neighboring neurons of the same ganglion are stimulated repetitively [57]. This implicates that peripheral tissue inflammation and sensitization can induce DRG neuronal cross-depolarization coupling that adjacent neurons activate together contributing to pain hypersensitivity, but this rarely happens in naive animals [40]. And this coupling activation occurs among various sized neurons including small-diameter nociceptors and large-diameter low-threshold mechanoreceptors [40]. Therefore, although MIA produces DRG with co-mingled sensitized and “normal” axons, damaged neurons can induce an increase of pronociceptive ion channel activity in both damaged neurons and adjacent intact neurons, leading to similar electrophysiological changes and increased discharge frequency in the damaged and neighboring intact PSNs. Additionally, microglial mechanisms contribute to OA-pain as in neuropathic pain [58], and the activated microglia can sense and regulate neuronal activity, which is expected to affect all populations of PSNs, since the highly proliferated microglia can be detected in wrapping most of the PSNs in DRG ipsilateral to MIA injection [14, 16]. Together, PSNs in OA pain can be extensively hypersensitized and convert the sensitized signals into action potentials, transmitted along PSN somata and afferents extending to peripheral terminals that decode dermatome sensitization and referred pain independent of joint damage, and to central terminals that relay the painful stimuli to the spinal DH and then to higher levels of the central nervous system neuraxis where they are decoded as conscious pain responses.

DRG-targeted pain interventions, such as DRG stimulation [44, 45] and pronociceptive molecule knockout in PSNs [43, 59], have been proved to be effective in mitigating OA pain behavior. The success of these DRG-targeted interventions gives us the insight to rationalize that AAV-mediated block of PSN excitability can be an alternative approach to effectively mitigate OA pain behavior. Here, the results proved that AAV-mediated DRG-targeted block of sensory neuronal T-type/Ca_V_3.2 activity succeeded in attenuating neuropathic pain behavior in a rat model of MIA-OA pain. Small molecule inhibitors selective for Ca_V_3.2 have been shown to produce dose-dependent antinociception in rat MIA-induced knee-joint pain [60] and neuropathic pain [61]. However, the widespread expression and multitude functional processes controlled by T-type/Cav3.2 have complicated the use of blockers for chronic pain [62]. For clinical analgesia, the peripheral sensory nervous system (PSNS) is a particularly accessible site for devising new treatments, especially the DRG-PSNs which initiate nociception and have a central role in the development and maintenance of painful neuropathy [63]. Delivering drugs to the PSNS is well developed and safe, for instance, as used in clinical anesthesia for regional blockade and by pain physicians for diagnosis and treatment of radiculopathy [64]. Injection into the DRG has minimal consequences in preclinical models [22]. It has also been demonstrated that unintentional intraganglionic injection commonly accompanies clinical foraminal epidural steroid injection [64], a very common procedure with minimal risk of nerve damage. Thus, the PSNs are particularly suitable for targeting new analgesic treatments, especially at the level of the associated pathological DRG.

This study is formulated to test the effectiveness of targeting PSN-T-type/Cav3.2 for OA-pain relief. A limitation is that a MIA-OA model is used in our current study, which can be differentiated in pain pathogenesis from human OA pain; however, features of MIA-induced disease is relevant to some aspects of human OA [65], including the knee damages and neuropathic pain-like symptoms. Additionally, whether DRG-targeted approach would prevent the nociceptive signals from reaching the SDH needs to be further investigated. One caveat of the present study is that therapeutic AAV vector is selectively tropism to PSNs with minimal glial tropism. Given the increasing evidence that DRG reactive microgliosis play a critical role in the development of OA pain [14], microglia and related signaling molecules hold promise as targets for OA pain control. Ideally, the development of AAV vector that carries chimeric peptide aptamers or aptamer-siRNA chimeras capable of interfering with multiple dysfunctional nociceptive hubs, as well as activated microglia, may hold substantial value as a therapeutic strategy for relieving refractory OA chronic pain.

## Conclusions

Our data provide preclinical relevance showing that suppression of T-type/Ca_V_3.2 channels selectively in the PSNs may be a potential approach to intervene in chronic OA pain behavior. It is necessary to validate the therapeutic potential of this strategy in other OA models in future investigations.

## Supplementary Information

**Additional file 1: Figure S1**. MIA OA knee histopathology. H&E stained sagittal section of saline-injected knees, showing full-depth normal cartilage and normal subchondral bone structure in male (A) and female (C). OA-like findings in the representative H&E-stained sagittal sections of knee 8 weeks after MIA (2mg) injection, showing articular cartilage loss (arrowheads), reduced chondrocyte numbers, subchondral bone collapse (arrows) in both male (B) and female (D). Scale bar: 500 μm for all. **Figure S2**. Analgesia of MIA-OA pain by DRG delivery of AAV6-3.2iPA1 (male rats). Analogous figures (A-F) to Fig. 1 for treatment with AAV6-3.2iPA2 shows comparable effectiveness to MIA-OA pain likewise to AAV6-3.2iPA2. Representative IHC montage images show double immunostaining of Tubb3 (red) and GFP-3.2iPA1 (green) (G). GFP-3.2iPA1 signal (green) is not detected in GFAP-positive glial cells (H, red). Scale bar: 100μm for all images.

## Data Availability

All experimental data generated or analyzed in this study are either included in this article (and its supplementary information files) or will be made available upon reasonable request.

## Abbreviations

OA: Osteoarthritis
MIA: Monosodium iodoacetate
DRG: Dorsal root ganglia
PSN: Primary sensory neuron
vF: von Frey
CPP: Conditioned place preference
GBP: Gabapentin
AAV: Recombinant adeno-associated viruses
Cav3.2: T-type calcium channel 3.2α1H
3.2iPA: Cav3.2 inhibitory peptide aptamer
3.2NP: Cav3.2 N-terminal inert peptide
GFP: Green fluorescent protein
AP: Action potential
RMP: Rest membrane potential
