# Supplementary material for: Selective block of sensory neuronal T-type/Cav3.2 activity mitigates neuropathic pain behavior in a rat model of osteoarthritis pain
Source: Arthritis Res Ther. 2022 Jul 16;24:168. doi: 10.1186/s13075-022-02856-0 (PMC9287929; doi:10.1186/s13075-022-02856-0)
Supplement: Supplementary file 1 — Additional file 1: Figure S1. MIA OA knee histopathology. H&E stained sagittal section of saline-injected knees, showing full-depth normal cartilage and normal subchondral bone structure in male (A) and female (C). OA-like findings in the representative H&E-stained sagittal sections of knee 8 weeks after MIA (2mg) injection, showing articular cartilage loss (arrowheads), reduced chondrocyte numbers, subchondral bone collapse (arrows) in both male (B) and female (D). Scale bar: 500 μm for all. Figure S2. Analgesia of MIA-OA pain by DRG delivery of AAV6-3.2iPA1 (male rats). Analogous figures (A-F) to Fig. 1 for treatment with AAV6-3.2iPA2 shows comparable effectiveness to MIA-OA pain likewise to AAV6-3.2iPA2. Representative IHC montage images show double immunostaining of Tubb3 (red) and GFP-3.2iPA1 (green) (G). GFP-3.2iPA1 signal (green) is not detected in GFAP-positive glial cells (H, red). Scale bar: 100μm for all images. [file 13075_2022_2856_MOESM1_ESM.docx]

**Supplementary Information**

# Selective block of sensory neuronal T-type/Cav3.2 activity mitigates neuropathic pain behavior in a rat model of osteoarthritis pain

Brandon Itson-Zoske, Seung Min Shin, Hao Xu, Chensheng Qiu, Fan Fan, Quinn H. Hogan, and Hongwei Yu


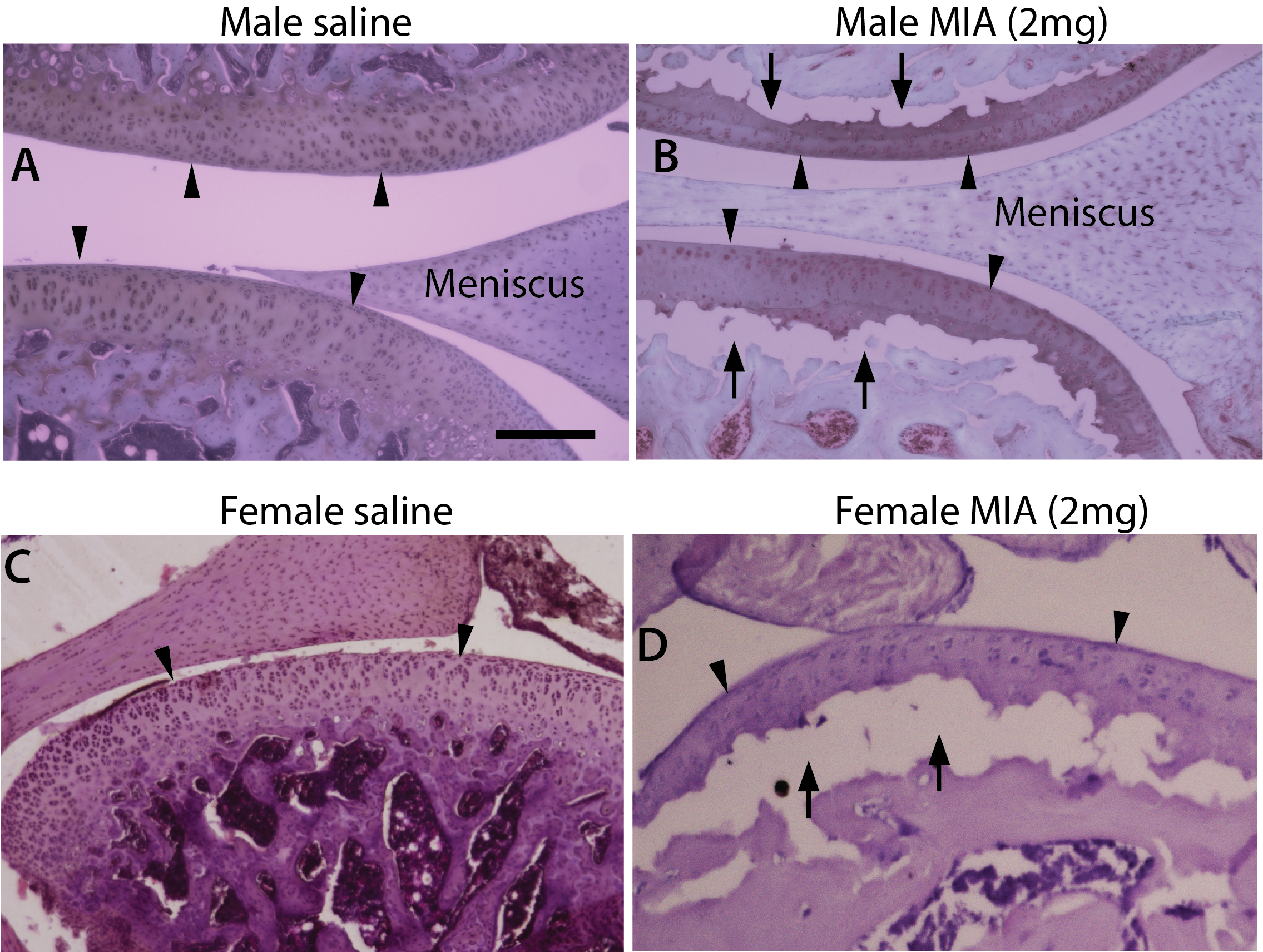


**Figure S1.** **MIA OA knee histopathology.** H&E stained sagittal section of saline-injected knees, showing full-depth normal cartilage and normal subchondral bone structure in male (**A**) and female (**C**). OA-like findings in the representative H&E-stained sagittal sections of knee 8 weeks after MIA (2mg) injection, showing articular cartilage loss (arrowheads), reduced chondrocyte numbers, subchondral bone collapse (arrows) in both male (**B**) and female (**D**). Scale bar: 500 µm for all.


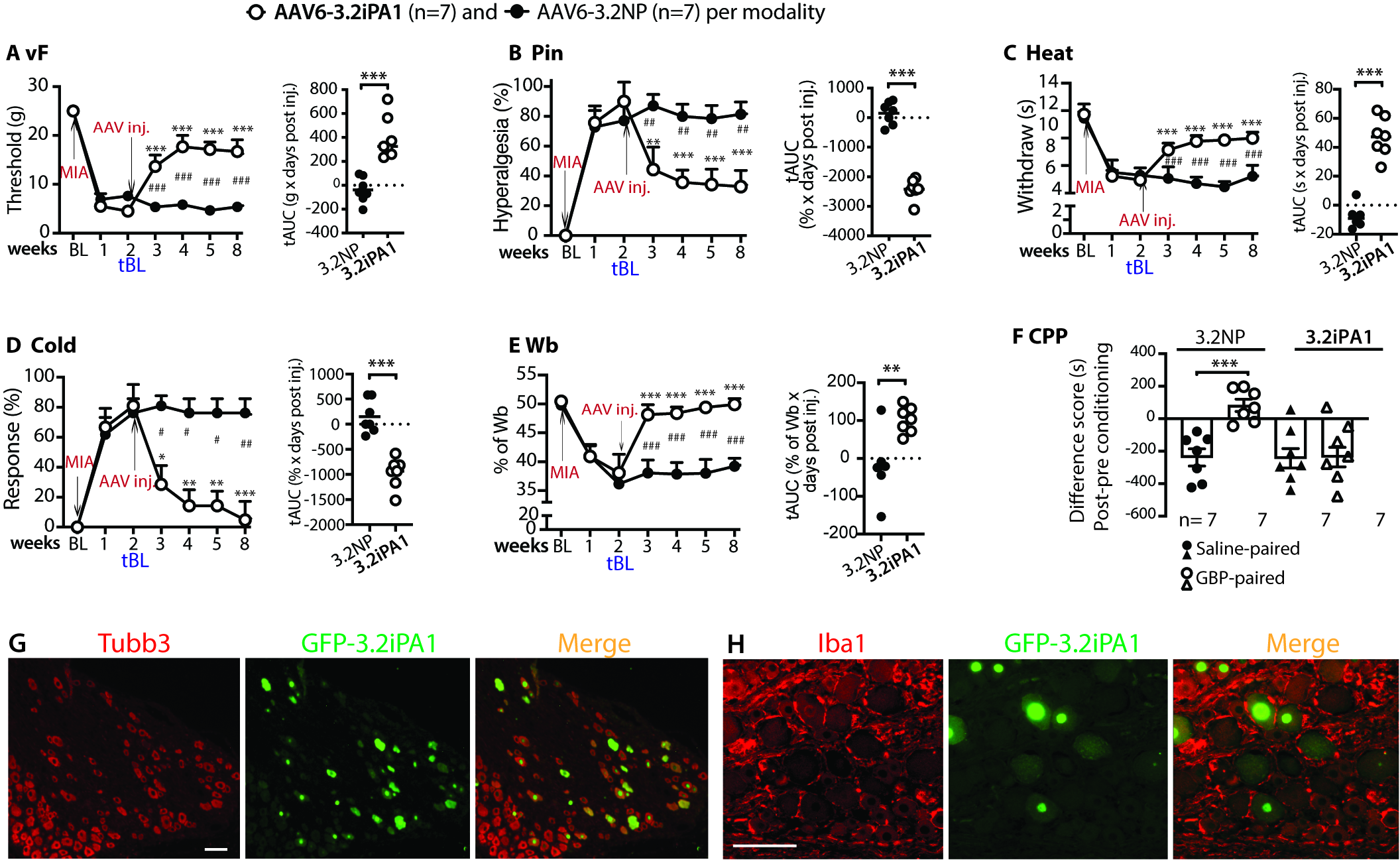


**Figure S2**. **Analgesia of MIA-OA pain by DRG delivery of AAV6-3.2iPA1 (male rats)**. Analogous figures (**A**-**F**) to Figure 1 for treatment with AAV6-3.2iPA2 shows comparable effectiveness to MIA-OA pain likewise to AAV6-3.2iPA2. Representative IHC montage images show double immunostaing of Tubb3 (red) and GFP-3.2iPA1 (green) (**G**). GFP-3.2iPA1 signal (green) is not detected in GFAP-positive glial cells (**H**, red). Scale bar: 100μm for all images.
